# Renal Function, Adherence and Quality of Life Improvement After Conversion From Immediate to Prolonged-Release Tacrolimus in Liver Transplantation: Prospective Ten-Year Follow-Up Study

**DOI:** 10.3389/ti.2022.10384

**Published:** 2022-12-19

**Authors:** Luca Toti, Tommaso Maria Manzia, Francesca Blasi, Ilaria Lenci, Leonardo Baiocchi, Nicola Toschi, Giuseppe Tisone

**Affiliations:** ^1^ HPB and Transplant Unit, Department of Surgery, University of Rome Tor Vergata, Rome, Italy; ^2^ Hepatology and Liver Transplant Unit, University of Rome Tor Vergata, Rome, Italy; ^3^Department of Biomedicine and Prevention, University of Rome Tor Vergata, Roma, Italy; ^4^ Athinoula A. Martinos Center for Biomedical Imaging, Harvard Medical School, Boston, MA, United States

**Keywords:** liver transplantation, immunosuppression, quality of life, Tacrolimus, adherence

## Abstract

Immunosuppression non-adherence is a major cause of graft failure after liver transplantation. The aim of this study was to evaluate practice surrounding conversion from immediate-release to prolonged-release Tacrolimus formulation and to assess patient adherence and quality of life (QoL). One hundred and seven adult liver transplant recipients, receiving immediate-release Tacrolimus for a minimum of 6 months, were converted to prolonged-release formulation, based on a dose ratio of one (1:1). The median follow-up was 120 [IQR, 120–123] months. Tacrolimus dosage and blood level, liver and renal function, lipid and glucose profiles were recorded. In addition, questionnaires were submitted to evaluate adherence and QoL following conversion. No rejection was recorded. The median serum Tacrolimus blood level decreased over 1 month (5.80, [IQR, 2.0–10.8] vs. 3.8 [IQR, 1.4–8.7]; *p* < 0.0005). Significant improvement in renal function was noted (median GFR was 81.7 [IQR, 43.4–128.6] vs. 73.9 [IQR, 27.1–130.2]; *p* = 0.0002). At the end of the follow-up, conversion resulted in an overall decrease in non-adherence of 53.3% (*p* = 0.0001) and an improvement in QoL was reported by 76.2% of patients. Thus, 1:1 conversion from immediate to prolonged-release Tacrolimus is safe, feasible and efficient, avoiding under-therapeutic and toxic peak concentrations, improving renal function, adherence to immunosuppression and overall patient QoL.

## Graphical Abstract

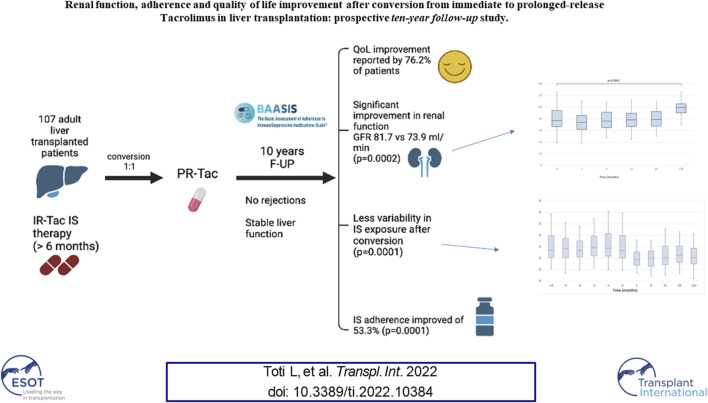

## Introduction

In recent decades, the introduction of new immunosuppressive drugs has contributed to graft survival in solid organ transplantation, decreasing the incidence of acute rejection and thus improving patient survival and quality of life (QoL). However, immunosuppression (IS) has several side-effects including renal failure, infections, cardiovascular diseases, metabolic disorders and *de novo* malignancies (1-4). In addition, patients are required to follow a complex IS regimen, which includes multiple drugs and personalized daily dose schedules. This therapeutic complexity is often poorly tolerated by patients and is the main cause of non-adherence after solid organ transplantation (5-7), which is estimated at between 15 and 55% (8-10). Therapeutic complexity is also the leading cause of preventable graft loss (4, 11-13). Therefore, simpler treatment regimens, such as once-daily dosing, have been suggested to help improve adherence in transplant recipients (14, 15). Furthermore, prolonged-release formulations may increase safety profiles avoiding toxic peaks and under therapeutic concentrations, which are observed in narrow therapeutic index drugs, including Tacrolimus (Tac) (16, 17).

Tac is frequently used in liver transplantation (LT). In addition to the immediate-release formulation (IR-Tac, Prograf^®^; Astellas Pharma US, Inc., Deerfileld, IL, USA), administered twice daily to maintain stable blood levels, a prolonged-release (PR-Tac, Advagraf®, Astellas Pharma Europe BV, Netherlands) formulation was licensed in Europe in 2007 for the prevention and treatment of graft rejection. Conversion from IR to PR-Tac has been studied in maintenance LT recipients (18-21), and the pharmacokinetic of IR-Tac and PR-Tac has been shown to be significantly different.

The main aim of this study was to explore tolerability and safety after conversion from IR to PR-Tac in adult LT patients. Secondary endpoints were patient adherence and QoL. Third endpoints were to evaluate the changes in concentration/dose ratio (C/D), C/D intra-patient variability following conversion from IR to PR-Tac, based on a dose ratio of 1 (1:1).

## Materials and Methods

This is a prospective, single arm study and patients were followed up at one, six, 12, 60 and 120 months between December 2010 and March 2021 in our hospital.

### Inclusion Criteria

All adult patients, who underwent LT, who were on IR-Tac-based IS regimen for at least 6 months, with stable liver function test (LFT) and serum creatinine levels <2.0 mg/dl were enrolled in this study.

### Exclusion Criteria

Patients were excluded in case of pregnancy, breastfeeding, malignancy, severe systemic infection requiring any therapy that could modify Tac pharmacokinetics, or the use of any other investigational drugs.

### Standard Immunosuppression Management

In general, the IS therapeutic protocol of our centre requires that corticosteroids are not used unless the patient has autoimmune pathologies. In patients with stable liver function, Tac monotherapy is usually achieved 1 year after the transplantation (22).

### Conversion Protocol to PR-Tac

The conversion from IR-Tac to PR-Tac started as soon as the new formulation was available in our hospital. All patients enrolled in the study were switched to PR-Tac, individually, during a 2 months period, and they were followed-up for at least 10 years. The starting dose of PR-Tac was exactly the same as the dose of IR-Tac taken by the patient at the time of conversion (1: 1).

Tac levels were measured in our central laboratory using a high-performance liquid chromatography-mass-spectrometry procedure (23). Patients were closely monitored during the study and Tac doses were adjusted to maintain adequate blood levels to maintain normal liver function and preventing rejection.

### Clinical and Biomedical Parameters

At follow-up, physical examination and measurement of vital signs were performed, contingent adverse events were noted, and laboratory test results were checked. Arterial hypertension was defined as systolic blood pressure >140 and/or diastolic >90 mm Hg at two subsequent visits or when antihypertensive treatment was prescribed. Diabetes mellitus was defined as fasting glucose >126 mg/dl at two subsequent visits or when hypoglycemic treatment was used. Dyslipidemia was defined as cholesterolemia >220 mg/dl and/or triglyceridemia >200 mg/dl at two subsequent visits or when using hypolipidemic treatment.

LFTs including aspartate aminotransferase (AST), alanine aminotransferase (ALT), gamma-glutamyl-transferase and bilirubin were performed at each clinic. Elevated transaminases, defined as twice the upper limit of our laboratory cut-off (AST >68U/L; ALT >110U/L), triggered closer surveillance and a liver biopsy when LFT abnormalities persisted (24). Graft loss was defined as retransplantation or death. Renal function was assessed using the glomerular filtration rate (eGFR, Modification of Diet in Renal Disease formula, MDRD) formula.

### Adherence and Quality of Life

Adherence was assessed using the ‘‘Basel Assessment of Adherence Scale to Immunosuppressives” (25) (BAASIS) questionnaire. This tool consists of a four-item validated questionnaire and a Visual Analog Scale (VAS). The first part addresses adherence including timing, missed dose and a “drug holiday” defined as >24 h interval between two consecutive doses. The VAS is a 100-point score, where patients report adherence in the previous 4 weeks from 0 to 100 (drug therapy never/always taken as prescribed) thereby assessing adherence as a continuous variable. The BAASIS form was completed by patients once pre-conversion to assess adherence to IR-Tac formulation and again at one- and ten-years following conversion to assess adherence to PR-Tac formulation and tolerability over time.

A *de novo* questionnaire (unpublished data) was developed to address those aspects of the IS regimen that influence patient care and the general perception of good or poor QoL in patients taking PR-Tac versus IR-Tac. The questionnaire was designed to be short, simple and easy to understand, to ensure a high completion rate with minimal missing data. The questionnaire, filled in anonymously by patients, collected demographic information (age, gender, and marital and employment status), and included three additional questions. Demographics were collected to facilitate the interpretation of the data at the end of the study. The first question was completed in the pre-conversion phase and queried the possible difficulty of taking multiple daily doses of drugs using a binary response option (YES/NO). The response was followed by a four-point Likert scale measuring the degree of difficulty, with two positive (very, quite) and two negative (little, very little) quantitative responses. At 12 and 120 months, questions two and three were administered. The second question assessed the possible satisfaction of the new drug regimen using a binary response option (YES/NO). The third question evaluated the perception of an improvement in QoL following the intake of the single-dose drug with a four-point Likert scale measuring the degree of improvement with two positive (very, quite) and two negative (little, very little) quantitative responses.

A positive response to the first question assumed dissatisfaction with taking multiple daily drugs, which was confirmed by positive responses on the Likert scale. Positive responses to questions 2 and 3 indicated satisfaction with the new therapeutic regimen and increased perception of QoL.

### Statistical Analysis

Data were presented as means (standard deviation), medians (interquartile range; IQR), or frequencies (percentage) as appropriate. For adherence data, categorical variables collected during follow-up were compared to baseline values using Fisher’s exact-test, while continuous data were compared to baseline values using the paired Student’s t-test. To simultaneously subject-wise as well as time-related changes, and possible interactions between them, all other variables were analyzed using multivariate linear mixed models modelling timepoints as a repeated within-subject factor and employing an unstructured estimate of the covariance matrix. As opposed to general linear models, mixed models have the advantage of being able to account for heterogeneous distances between timepoints, missing data as well as unequal variances and covariances. To account for possible confounding due to inter-patient variability, all models included gender, categorized disease etiology, time between therapy inception and conversion, and Tac blood levels (primary endpoint only) as covariates of interest.

Whenever a statistically significant (*p* < 0.05) overall effect of time was found, pairwise comparisons between timepoints were performed and corrected for multiple comparisons across pairs of timepoints using the Dunn–Šidák procedure.

Written informed consent was obtained from each patient prior to enrolment, without any patient refusing to participate in the study.

This study was conducted in accordance with the Declaration of Helsinki and approved by an Independent Ethics Committee prior to implementation.

## Results

One hundred and seven Caucasian adult LT recipients with a median age of 55 (IQR, 48–61.5) years were enrolled into the study. The median time from LT to study enrolment was 55 (IQR, 31–81) months. Patient characteristics are summarized in Table 1.

**TABLE 1:** Patient baseline characteristics.

Characteristic (N = 107 patients)	Median/[IQR] or no. (%)
Sex, Males	69 (64.5%)
Age at conversion (years)	55 [48–61.5]
Time from IR-Tac to conversion (months)	55 [31–81]
Indication for LT	
Hepatitis C virus	27 (25.2%)
Hepatitis B virus	24 (22.4%)
Alcohol	12 (11.2%)
HCC	29 (27.1%)
Other	15 (14.1%)
Weight at baseline, kg	69 [62–75]
Comorbidity	
Diabetes mellitus	14 (13.1%)
Hypertension	21 (19.6%)
Hyperlipidemia	26 (24.3%)
Renal impairment (eGFR <60 ml/min/1.73 m^2^)	14 (13.1%)

### Primary Endpoint (Tacrolimus Tolerability and Safety)

At enrolment, 74 patients (69%) were on IR-Tac monotherapy. Median Tac daily dose was 2.0 (IQR, 1.5–3.0) mg and similar values were reported within the first 12-month after conversion. Eight-six out of 107 (81.9%) patients continued with the same Tac dosage after 1 year and the dosage was decreased in six patients (5.7%), increased in 11 patients (10.5%) and two (1.9%) patients were withdrawn from PR-Tac: the first patient due to frequent episodes of hypertension, diarrhea and vertigo and reconverted to IR-Tac; the second one due to *de novo* intestinal adenocarcinoma and subsequently switched to mTOR-inhibitor monotherapy.

By the end of the follow-up, 91 (85%) patients were still on the PR-Tac IS regimen: 56 patients (52.3%) were maintained on the same dosage as baseline; 24 patients (22.4%) had their dosage decreased and 11 patients (10.3%) increased. Six (5.6%) were converted to a different IS drug between 12th and 120th month. Three patients (2.8%) initiated single drug treatment with mycophenolate mofetil due to blood hypertension at 28, 56 and 68 months respectively. Three patients (2.8%) were converted to mTOR-inhibitor due to HCC recurrence, breast cancer and colon adenocarcinoma at 58, 89 and 112 months, respectively. A total of eight (7.4%) patients died due to a cardiovascular accident (*n* = 4) or malignancy (*n* = 4; lung cancer (*n* = 2) and esophagus cancer (*n* = 2)) (Table 2).

**TABLE 2:** Tacrolimus dosage modifications at 12 and 120 months.

Overall to IR-Tac	12 months	120 months
No modification	86 (80.4%)	56 (52.3%)
Decreased	6 (5.7%)	24 (22.4%)
Increased	11 (10.5%)	11 (10.3%)
Converted to another drug	2 (1.9%)	8 (7.5%)
Deaths	—	8 (7.5%)

**FIGURE 1:**
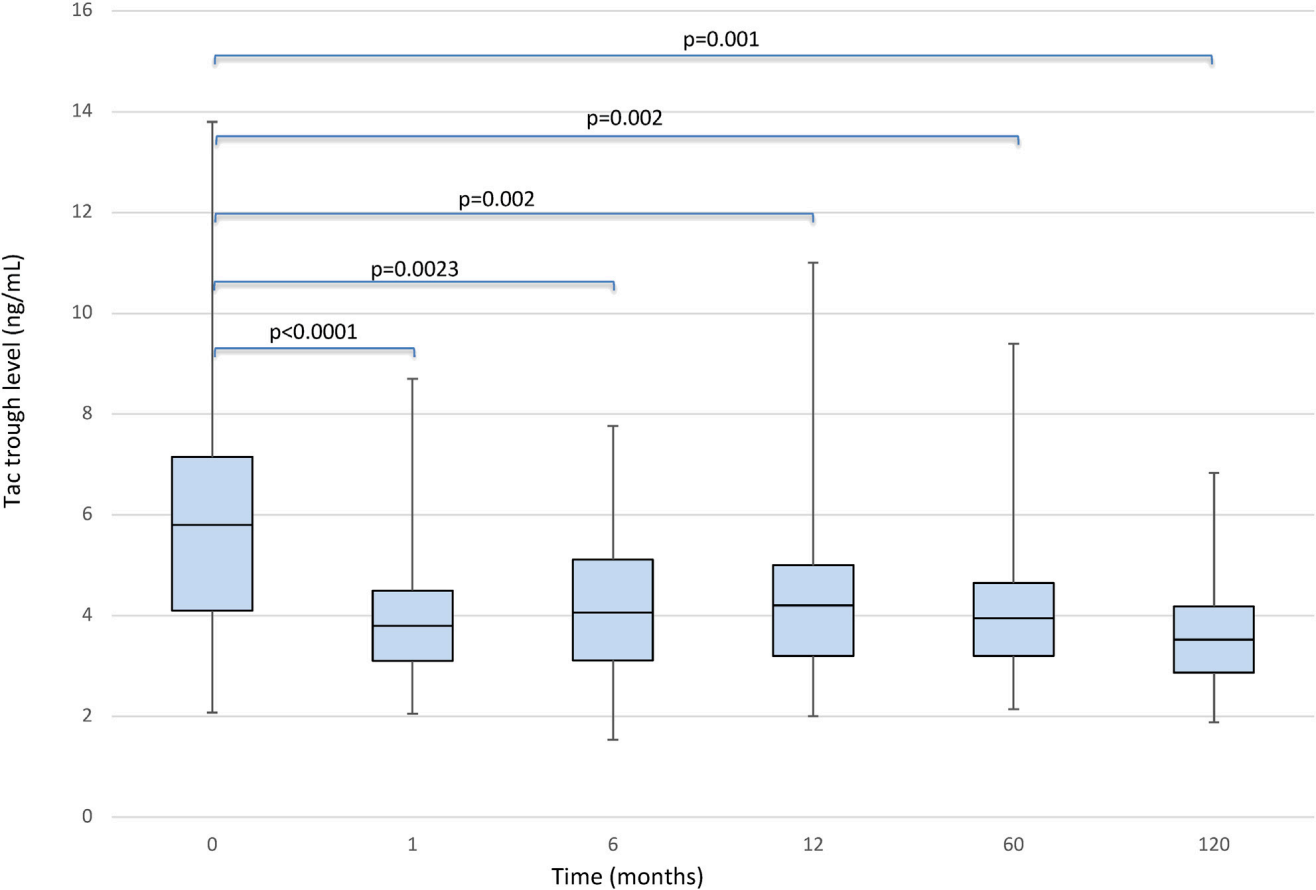
Boxplots showing median, interquartile range and 5th/95th percentile of Tacrolimus trough level as a function of timepoint.

The median serum Tac blood levels were 5.80 (IQR, 4.1–7.1) ng/ml and 3.80 (IQR, 3.1–4.5) ng/ml respectively (*p* < 0.0001) at baseline and 1 month after conversion, respectively. When the LFTs remained stable no dose adjustments were considered. In patients without dose adjustments, the median Tac blood levels remained 2.0 mg [IQR, 1.5–3.0] We found a significant effect of the timepoint factor (*p* < 0.0001) on Tac blood levels, which in post-hoc comparison appeared to be associated with the following differences: baseline vs. 1, 6, 60 and 120 months (1 m < baseline: *p* < 0.0001; 6 m < baseline: *p* = 0.023; 60 m < baseline: *p* = 0.002, 120 m < baseline: *p* = 0.001); 1-month vs. 6, 60 and 120 months (1 m < 6 m: *p* = 0.023; 1 m < 60 m: *p* = 0.003; 1 m > 120 m: *p* = 0.001); 6 months vs. 12, 60 and 120 months (6 m < 12 m: *p* = 0.01, 6 m > 60: *p* = 0.001, 6 m > 120 m: *p* = 0.001) (Figure 1).

No patient experienced clinical or biopsy-proven acute rejection (BPAR) after conversion. The 10-year survival was 92.6%.

**FIGURE 2:**
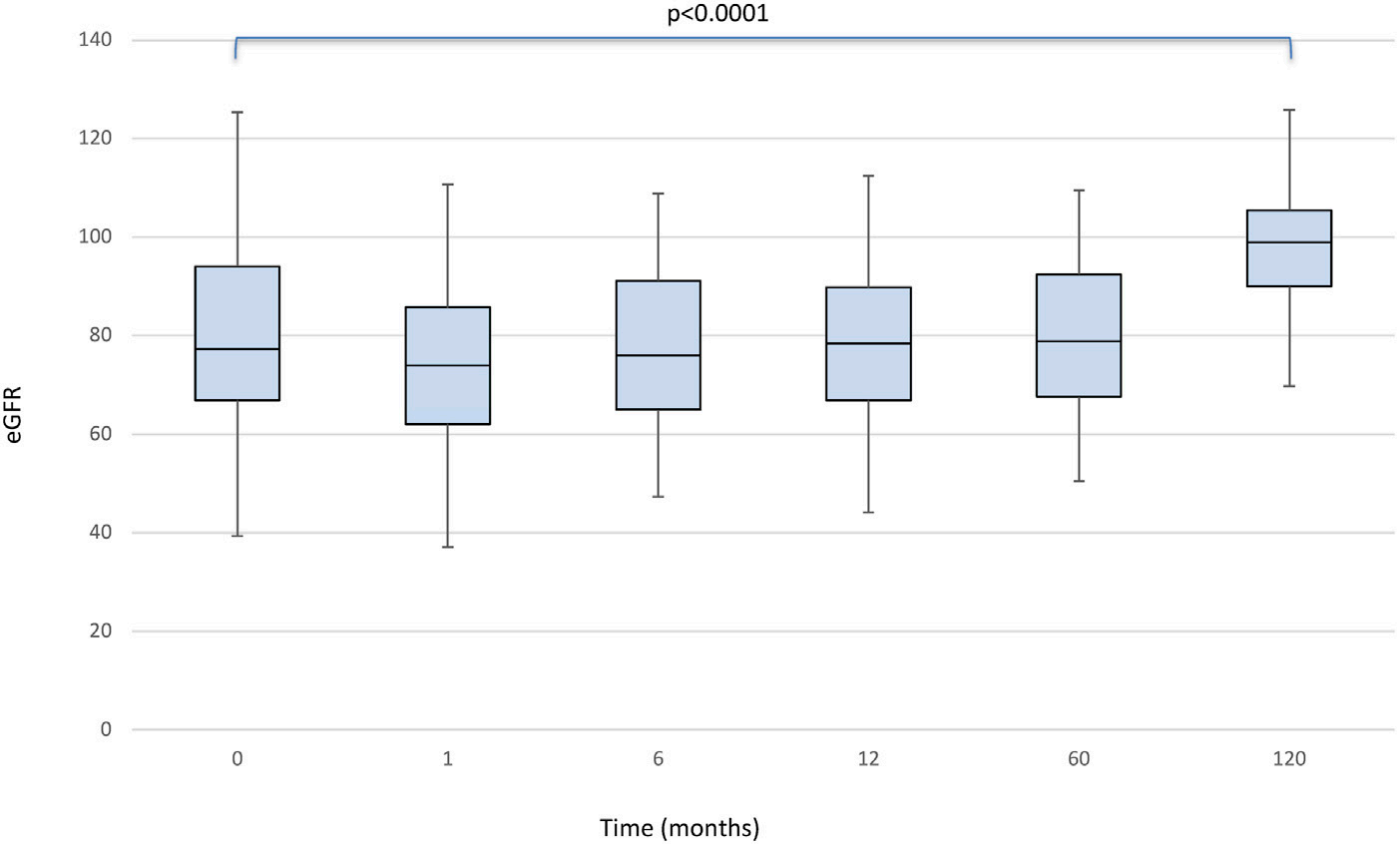
Boxplots showing median, interquartile range and 5th/95th percentile of eGFR as a function of timepoint.

There was no statistically significant effect for the timepoint factor on liver function. Even the comparison of glucose levels and cholesterol and triglycerides values between the pre and post conversion periods was not statistically significant. We found a significant effect of the timepoint factor (*p* < 0.0001) on eGFR (Figure 2), which in post-hoc comparison appeared to be associated with the following differences: baseline<120 months (*p* < 0.0001); baseline>1 month (*p* < 0.0001); baseline>6 months (*p* = 0.049); month 1<6, 12, 60 and 120 (all *p* < 0.001).

### Secondary End Points (Adherence and QoL)

The BAASIS questionnaire addressed different aspects of adherence, with the aim of identifying those areas where adherence has significantly increased. At baseline, 84 (78.5%) patients reported forgetting to take at least one drug dose in the previous 4 weeks, whereas this dropped to 24 (22.4%) and 27 (25.2%) patients at 12 and 120 months, respectively (*p* < 0.0001). Sixty-six patients (61.7%) declared at baseline that they had possibly missed two consecutive drug doses, which dropped to 12 (11.4%; *p* < 0.0001) and 22 (20.5%; *p* < 0.0001) patients at 12 and 120 months, respectively. Sixty-three patients (58.9%) did not respect the therapeutic intake time at baseline but this decreased to 15 (14%; *p* < 0.0001) and 25 (23.3%; *p* < 0.0001) patients at 12 and 120 months, respectively. Five patients (4.7%) admitted to taking lower drug dosages than medically prescribed, which dropped to three (2.8%; *p* = 0.72) and no (0%, *p* = 0.06) patients at 12 and 120 months, respectively.

Median VAS ratings of patient adherence were 90 (IQR,75–100) at baseline and were significantly higher at 12 months (97 [IQR, 85–100]; *p* = 0.0009) and 120 months (95 [IQR, 87–100]; *p* = 0.0008) (Table 3).

**TABLE 3:** Adherence evaluation at Baseline, 12 and 120 months by BAASIS and VAS.

BAASIS	Baseline (*n* = 107)		Follow-up at 12 months (*n* = 105)		*p*-value**	Follow-up at 120 months (*n* = 91)		*p*-value**
	N (%)		N (%)			N (%)	
ITEM 1: Dose not taken	84 (78.5%)		24 (22.8%)		0.0001	23 (25.2%)		0.0001
ITEM 2: Consecutive doses not taken	66 (61.7%)		12 (11.4%)		0.0001	19 (20.9%)		0.0001
ITEM 3: Dose taken with delay	63 (58.9%)		15 (14.3%)		0.0001	21 (23.1%)		0.0001
ITEM 4: Dose auto-reduced	5 (4.7%)		3 (2.8%)		0.7214	-		0.0634
Overall Adherence*	17 (15.9%)					62 (68.1%)		0.0001
**VAS**	**Median**	**IQR**	**Median**	**IQR**	*p*-value***	**Mean**	**IQR**	*p*-value***
SCALE 0-100	90	75–100	97	85–100	0.0009	95	87–100	0.0008

The three separate questions regarding QoL were completed by all participants at baseline. At baseline 66 (61.7%) patients indicated that they experienced difficulty with taking multiple doses of immunosuppressants daily, which was similar after 12 months (*n* = 69; 65.7%) and 120 months (*n* = 61; 67%). Filter questions showed that 40 (60.6%), 49 (71.0%) and 45 (73.8%) of patients found it very difficult to take more than one type of drug at baseline, 12 months and 120 months, respectively. Twenty-six (39.4%), 20 (72.4%) and 48 (78.7%) patients found it very difficult to take one or more doses of the same drug at baseline, 12 months and 120 months, respectively. Thirty (45.4%), 67 (97.1%) and 60 (98.4%) patients found it very difficult to take drugs at different times at baseline, 12 months and 120 months, respectively.

The second item addressed the degree of satisfaction with the PR-Tac therapeutic regimen. The data showed that 98 (93.3%) and 79 (86.8%) patients were very satisfied at 12 and 120 months, respectively. The third item asked if there had been an improvement in QoL after conversion. Eighty (76.2%) and 75 (82.4%) patients confirmed that their QoL had improved at 12 and 120 months respectively, with the filter question showing that 82.5% and 86.8% felt that QoL had very much improved at 12 and 120 months, respectively (Table 4).

**TABLE 4:** QoL questionnaire administered at 12 and 120 months: items 1, 2 and 3.

N	Item	Answer	Baseline N = 107	12 months *n* = 105	120 months *n* = 91
1	Do you consider it difficult to take two or more doses of immunosuppressant drugs during the day?	Yes	66 (61.7%)	69 (65.7%)	61 (67%)
		No	41 (38.3%)	36 (34.3%)	30 (33%)
	• Take one or more types of drugs	Very difficult	40 (60.6%)	49 (71.0%)	45 (73.8%)
		Average	26 (39.4%)	20 (29.0%)	16 (26.2%)
		Easy	—	—	—
	• Take one or more tablets for type of drug	Very difficult	26 (39.4%)	50 (72.4%)	48 (78.7%)
		Average	40 (60.6%)	19 (27.5%)	13 (21.3%)
		Easy	3 (6.54%)	—	—
	• Take the drug at different times	Very difficult	30 (45.4%)	67 (97.1%)	60 (98.4%)
		Average	31 (47.0%)	2 (2.9%)	1 (1.6%)
		Easy	5 (7.6%)	—	—
2	Indicate the degree of satisfaction of the new regimen of taking the drug	Very satisfying	—	98 (93.3%)	79 (86.8%)
		Average	—	1 (0.9%)	10 (11.0%)
		Unsatisfactory	—	1 (0.9%)	—
		Indifferent	—	5 (4.9%)	2 (2.2%)
3	Do you feel an improvement in the quality of your life?	Yes	—	80 (76.2%)	75 (82.4%)
		No	—	25 (23.8%)	16 (17.6%)
	• Indicate how much your life has improved	Very much	—	66 (82.5%)	79 (86.8%)
		Average	—	10 (12.5%)	11 (12.1%)
		Very little	—	4 (5.0%)	1 (1.1%)

### Third Endpoint (C/D Ratio and Intra-patient Variability)

**FIGURE 3:**
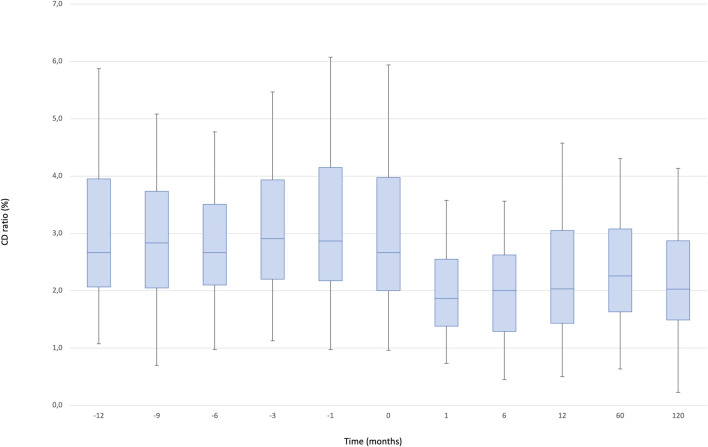
Boxplots showing median, interquartile range and 5th/95th percentile of CD/ratio as a function of timepoint.

The median C/D ratio at baseline was 2.67 (IQR, 2.7–4.0). After one and 12 months, the ratio decreased to 1.87 (IQR, 1.9–2.6) and 2.03 (IQR, 2.0–2.6), respectively and remained stable during the follow up (2.03 [IQR, 2.0–2.9]: *p* < 0.000001) (Figure 3).

We observed a significant effect of the timepoint factor (*p* < 0.0001) associated with the following differences: 12 months vs. baseline, 1, 6, 60, 120 months (12 m < baseline: *p* = 0.0001, 12 m > 1 m: *p* = 0.001, 12 m > 6 m: *p* = 0.007, 12 m < 60 m: *p* = 0.001, 12 m > 120 m: *p* = 0.053), 6 months vs. baseline, 1, 12, 60, 120 months (6 m < baseline, 6 m > 1 m, 6 m < 12 m, 6 m < 60 m, 6 m < 120 m; all *p* = 0.0001), 1 month vs. baseline, 6, 12, 60, 120 months (1 m < baseline, 1 m < 6 m, 1 m < 12 m, 1 m < 60 m, 1 m < 120 m; all *p* = 0.0001), 120 months vs. baseline, 1, 6, 12, 60 months (120 m < baseline, 120 m > 1 m, 120 m > 6 m, 120 m < 12 m, 120 m < 60 m; all *p* = 0.0001). In addition, we compared 10 consecutive pre-conversion timepoints to 10 consecutive post-conversion measurements to evaluate the change of Tac blood levels and dose in a long-term observation: the mean C/D ratio was significantly higher pre-conversion compared to post conversion (3.29 [IQR, 2.7–4] versus 2.58[IQR, 2.3–2.9]: *p* = 0.008), while the coefficient of variation of the C/D ratio was significantly lower pre-conversion compared to post-conversion (2.12 versus 1.19: *p* = 0.003).

## Discussion

IR-Tac was considered a pillar of immunosuppressive therapy for solid organ transplantation for nearly 20 years, with excellent protection against organ rejection. Many studies have evaluated the effectiveness of converting from IR-Tac to PR-Tac, the latter able to facilitate adherence, to improve the QoL of transplant recipients and consequently their long-term results. (26, 27). In 2005, Florman et al. (6) reported the first conversion pharmacokinetics for stable LT recipients, concluding that the steady-state Tacrolimus exposure of PR-Tac was equivalent to IR-Tac after conversion on a milligram-for-milligram basis in stable LT recipients.

In our study the Tac blood levels decreased following conversion in 76% of cases and remained stable to the end of follow-up, which is similar to previously reported studies (19, 28).

An important aspect of this study is the side-effect profile of the anti-rejection drugs correlating to tac blood levels. The initial phase showed a decrease in Tac blood levels over time. Despite this, graft function remained stable with good function and was maintained over time with less side-effects. Time impacted significantly on serum Tac blood levels, which dropped sharply in the early post-conversion period.

With normal aging, nephron loss occurs and is detectable to some extent by the age-related decrease in eGFR (29, 30). Several studies that have analyzed the deterioration of renal function with increasing age in the healthy population show that eGFR shows a physiologic decrease between 0.3 and 1.4 ml/min/1.73 m^2^/year (31, 32). In addition, calcineurin inhibitors, widely recognized as the mainstay of IS used to prevent graft rejection, have an important nephrotoxic side-effect profile. The expected gradual reduction in eGFR in LT recipients is the result of different mechanisms including immunologically mediated damage concurrent to the IS side-effects, nephrotoxicity and the development of cardiovascular risk factors (29, 33). However, a significant improvement in renal function was seen in our study: using eGFR, a significant effect of the timepoint factor (*p* < 0.0001) was seen, with a retrospective comparison showing the following differences at baseline vs. 1 month (*p* < 0.0001), baseline vs. 6 months (*p* = 0.049), month 1 vs. 6, 12, 60, and 120 (all *p* < 0.001). There were no new cases of posttransplant diabetes or glucose intolerance or any increase in adverse events associated with Tacrolimus use after conversion to PR-Tac.

It can be hypothesized that extended-release Tac may influence drug absorption and avoid drug peaks whilst maintaining adequate drug blood levels to avoid rejection.

Self-reporting adherence instruments having a tendency to overestimate adherence and under-report non-adherence due to increased awareness and pleasing the physician. However, BAASIS is considered a valid tool as it uses a rigorous definition of non-adherence, classifying a patient as non-adherent in case of positive answer to any of the four questions to be given. Non-adherence at study entry was considerably high especially regarding the evening dose, which has also been found by previous studies (6,34). At the end of the follow-up, however, these high adherence rates increased even further. The data clearly demonstrate that simply reducing the number of daily doses positively influences adherence leading to improved compliance and patient satisfaction. The important improvement in adherence following conversion to PR-Tac formulation is also evidenced in previously published studies. (35-37)

Adherence rates improved significantly between baseline and the end of the study in terms of missing one or more doses, violating drug timing and autonomous prescription modification. Patients themselves felt more adherent at the end of the study with 97% of patients defining themselves as “adherent” at 120 months compared with 90% at baseline. Patients reported having difficulty taking more than one IS drug in 61.7% of cases before conversion, not knowing about PR-Tac, subsequently, 93.3% defining the PR-Tac regimen as “very satisfactory.” During medical interviews, 68% referred the evening dose as being the most difficult to self-administer for perceived interference with social life and sense of freedom. This has radically changed following conversion to PR-Tac.

Freedom of choice relating to time of drug administration directly impacted on perception of improvement in QoL for the majority of patients. QoL improvement was reported in over 82.4% of patients in our study following conversion. The reason why we decided to use a *de novo* questionnaire, drawn up and validated in collaboration with the Center for Psychology of our hospital, is due to the fact that in the literature, in our opinion, there were no validated questionnaires that had the characteristics, suitable for a complete evaluation of our patients which would allow us to obtain such complete results and which went hand in hand with the BAASIS, used for the assessment of adherence. The fact that no patient enrolled, with the sole constraint of taking IR-Tac for at least 6 months before signing the informed consent to the study, was for us surprising evidence of the excellent relationship of trust that we establish every day with all the people we follow in our post-transplant clinic and further confirmation of the patients’ desire to seek “simpler” therapy regimens to follow.

Despite the absence of a control group that may evidence bias, we have considered the group itself before conversion as satisfactory for comparison.

There is significantly less intrasubject variability in exposure after conversion to PR-Tac: the mean C/D ratio was significantly (*p* = 0.008) higher pre-conversion as compared to post conversion, while the coefficient of variation of the C/D ratio was significantly (*p* = 0.003) lower pre-conversion compared to post-conversion, indicating greater stability post-conversion compared to pre-conversion.

In conclusion, our study demonstrates that 1:1 conversion from IR-TAC to PR-TAC is safe, feasible, efficient, and well-tolerated. Hepatic and renal function was closely monitored and no major dose adjustment to correct low Tac blood levels were required. Stable LT patients can be successfully switched from IR-Tac to PR-Tac formulation without risk of acute rejection even in the short term. A simplified formulation of Tac can improve patient adherence and their QoL. Improvement of renal function is probably due to lower Tac blood level exposure.

## Data Availability

The original contributions presented in the study are included in the article/supplementary material, further inquiries can be directed to the corresponding author.

## Abbreviations

BAASIS, Basel Assessment of Adherence Scale to Immunosuppressives; C/D, concentration/dose ratio; IR-Tac, immediate-release Tacrolimus formulation; PR-Tac, prolonged-release Tacrolimus formulation; VAS, Visual Analog Scale.
